# Effect of MWNT Functionalization with Tunable-Length Block Copolymers on Dispersity of MWNTs and Mechanical Properties of Epoxy/MWNT Composites

**DOI:** 10.3390/polym14153137

**Published:** 2022-08-01

**Authors:** Jingwei Liu, Yunsheng Ye, Xiaolin Xie, Xingping Zhou

**Affiliations:** 1 Chongqing Key Laboratory of Nano-Micro Composite Materials and Devices, School of Metallurgy and Materials Engineering, Chongqing University of Science and Technology, Chongqing 401331, China; 2019004@cqust.edu.cn; 2 Key Laboratory of Material Chemistry for Energy Conversion and Storage, Ministry of Education, School of Chemistry and Chemical Engineering, Huazhong University of Science and Technology, Wuhan 430074, China; xlxie@mail.hust.edu.cn; 3 Department of Materials and Optoelectronic Science, National Sun Yat-sen University, Kaohsiung 000800, Taiwan

**Keywords:** epoxy, MWNTs, toughness, interface, regulation, block copolymer

## Abstract

The dispersion level of carbon nanotubes (CNTs) and interface design are two of the most crucial roles in developing the superior mechanical performance of polymer/CNT nanocomposites. In this work, a series of azide-terminated poly(glycidyl methacrylate)-block-poly(hexyl methacrylate) (PGMA-*b*-PHMA) copolymers with different PHMA chain lengths and similar PGMA chain lengths were grafted on the surface of multiwall carbon nanotubes (MWNTs). PHMA length changes significantly impact the grafting density and solubility in organic solvents of as-prepared block copolymer functionalized MWNTs(bc@*f*MWNTs). Then, the bc@*f*MWNTs were introduced to epoxy, and the resulted epoxy/bc@*f*MWNT composites show better mechanical properties than neat epoxy and epoxy/p-MWNT composites. The results suggest that longer PHMA chains cause the two competitive and opposing effects on the dispersion state and soft interface. On the one hand, the longer PHMA chains on the surface of MWNTs would afford higher deformation for the matrix and enhanced mobility for MWNTs because of the soft and flexible nature of PHMA, enhancing the energy dissipation during strain. On the other hand, as the length of PHMA extends, the dispersion level of bc@*f*MWNTs in epoxy declines, which is harmful to the composite’s mechanical properties. Hence, epoxy/bc@*f*MWNTs composites with relatively short PHMA chains show the best tensile and fracture properties.

## 1. Introduction

Epoxy is a good candidate in advanced technological fields as a thermoset resin because of its excellent mechanical and thermal performance [1]. However, due to the high crosslink density of the epoxy network [2], the shortage in toughness properties is one of the critical drawbacks in popularizing its use across numerous applications. The introduction of a second phase is a commanding approach to increasing the toughness of epoxy [3]. Different nanofillers, including CNTs, have been employed to improve epoxy toughness properties. Due to the requirement of effective load transfer, homogeneous dispersion of nanofillers is the prerequisite for the optimal performance of the nanocomposite [4].

It is well-known that the filler-matrix interfacial adhesion influences the composite strength. In contrast, the fracture toughness is more dependent on the interface structure between fillers and matrices [2]. The fracture toughness of epoxy/nanofiller composite cannot reinforce monotonically by simply improving dispersion state or interfacial adhesion [5]. Therefore, the functionalization of nanofillers, which could improve their dispersion level and radically enhance their interfacial interactions with the polymer matrix, is extremely important for enhancing their applications in epoxy composites [6,7]. Among the nanofillers’ surface functionalization strategies, silanization [8,9,10,11], carboxylation [12], and amidation [12,13] have been regarded as promising candidates for reinforcing the epoxy resin matrices due to their valuable features.

Due to the adequate solubilizing ability of polymer species, polymer functionalization of CNTs is an effective tool for improving the dispersity of nanotubes and modulating interfacial structure between CNTs and host polymers [14]. For example, a noticeable increase in tensile strength (45–75%) can be obtained by introducing 0.1~0.7 wt% MWNTs functionalized with polyacryloyl chloride [15] or polyaniline [16] in MWNTs-filled epoxies. At the same time, incorporating 0.5 wt% poly (ether ether ketone) [17] grafted MWNTs into epoxies improved the K*_IC_* by 38% and tensile strength by 22%, respectively. Furthermore, it was found that nanofillers with a soft interface and good adhesion with the epoxy host can effectively toughen epoxy while maintaining strength properties [18,19]. However, there is a lack of a quantitative relationship between polymer modification and the mechanical status of the composite. We believe that tunable interphases between the polymer host and the CNTs not only adjust the structure of interfaces, but should also promote load transfer such that the toughness and strength of composites can be optimized simultaneously.

Nevertheless, the lack of diversity and versatility of homopolymers is detrimental to achieving desired regulations on dispersity and interface structure properties of CNT in epoxy. On the contrary, block copolymers (BCs) have a marked ability to control the dispersibility and interfacial structure of fillers in the matrix by changing the monomer structures and molecular weight of two block polymers. Moreover, previous work demonstrated that block copolymers could effectively toughen epoxies with a slight decrease in the modulus [20] in CNT-based epoxy composites. Alternatively, it can be used as a non-covalent surfactant [21,22] in CNT-based epoxy and improve CNT dispersion of the CNTs in the system, as evidenced by an enhancement in fracture toughness without sacrificing the elastic modulus.

In this paper, the poly(glycidyl methacrylate)-block-poly(hexyl methacrylate) (PGMA-*b*-PHMA) with various chain lengths was employed to functionalize MWCNTs for toughening the epoxy resin by the following factors: (i) the outer block copolymer by poly glycidyl methacrylate (PGMA) containing epoxide groups can optimize the dispersion of MWNTs and strengthen interface bonding with the epoxy matrix; (ii) the inner block copolymer by polymethylmethacrylate (PHMA) is incompatible with epoxy resin and possesses a low *T*_g_ of −10~0 °C, initiating the interface deformation under loading; (iii) the well-controlled lengths in both PHMA and PGMA building blocks has a very high ability to regulate the properties of composites. To further tailor the epoxy/MWNTs interface structure, a series of block copolymers, consisting of poly (glycidyl methacrylate)-block-poly (hexyl methacrylate) with the same PGMA length and different PHMA lengths onto MWNTs, were subsequently introduced in the epoxy-based nanocomposites. The outer layer of PGMA provides a good dispersion of MWNTs and strong interface bonding with the epoxy, which is beneficial for increasing strength and modulus. In contrast, the introduction of inter-layered rubbery PHMA around MWNT provided a possibility to trigger cavitation and promote the local plastic deformation of the matrix to dissipate fracture energy. We believe that it is worth understanding how the chain length of interlayer PHMA will affect the dispersity of MWNTs and the mechanical properties of the resultant composites. To the best of our knowledge, this article first provides a quantitative relationship between functionalized molecular structure, filler dispersion, and composites’ mechanical properties for MWNTs-filled epoxy composites, which could offer a theoretical basis for the design and fabrication of epoxy-based composite materials.

## 2. Materials and Methods

### 2.1. Chemicals and Materials

Epoxy resin (diglycidyl ether of bisphenol-A based E-51 epoxy with an epoxide equivalent weight of 192) was purchased from Shanghai Resin Factory Co., China. The qualitative characteristics of epoxy resin are presented in Table 1. The hardener was composed of the methyl-hexahydro phthalic anhydride (MHHPA) and benzyl dimethylamine catalyst (BDA), with a weight ratio of 40:1. MHHPA and BDA were purchased from Shanghai Aladdin Biochemical Technology Co., Ltd. The qualitative characteristics of the hardener are presented in Table 2. The resin to hardener ratio equaled 1:0.82 for all nanocomposites. The pristine multi-wall carbon nanotubes, with a diameter of 15–20 nm and a length of 0.5–2 μm, were purchased from Chengdu Organic Chemicals Co. Ltd. The qualitative characteristics of MWCNTs are presented in Table 3. Hexyl methacrylate (HMA ≧ 98.5%) and glycidyl methacrylate (GMA, 98%), both from Aldrich, St. Louis, MO, USA were distilled before use. According to a previously published report [23], 3-azidopropyl 4-cyano-4-((phenylcarbonothioyl)thio) butanoate (Azido-CTAs) was synthesized. Before use, 2,2′-azobis-(2-methylpropionitrile) (AIBN, 98% from Aldrich, St. Louis, MO, USA) was recrystallized from ethanol twice and dried at room temperature under a vacuum. All other chemical reagents were used as received.

### 2.2. Synthesis of Block Copolymers

A series of azido-terminated PGMA-*b*-PHMA-N_3_ with nearby PGMA length and different PHMA lengths were prepared by reversible addition-fragmentation chain transfer (RAFT) polymerization. Firstly, PHMA with different PHMA lengths (denoted H1-H4) were synthesized in dioxane at 70 °C by using azido-terminated chain transfer agents (CTAs-N_3_). Then, the RAFT polymerizations of GMA were carried out by using PHMA-N_3_ (polymer H1 to H4) as macro-CTAs. The chain length of PGMA was controlled precisely by changing the feed ratio of monomers to CTAs.

### 2.3. Preparation of PGMA-b-PHMA@fMWNTs

In a typical experiment, a 250 mL flask was charged with MWNTs-Alkyne (200 mg) and DMF (60 mL). After stirring and sonicating for 30 min at room temperature, azide-functionalized PHMA-*b*-PGMA [PGMA-*b*-PHMA-N_3_; M_n (GPC)_ = 14,500 g mol^−1^] (1.7 g, 0.019 mmol), CuI (14 mg, 0.2 mmol), and DBU (1.5 g, 10 mmol) were added. The solution was deoxygenated by bubbling with nitrogen for at least 30 min. The flask was immersed in an oil bath preheated to 70 °C for 24 h. The solid was separated from the mixture by centrifugation. The collected solid was redispersed in THF (50 mL) and separated by centrifugation three times. The solid mass (PGMA-*b*-PHMA @*f*MWNTs) was dried at 60 °C overnight in a vacuum oven.

### 2.4. The Solubility of bc@fMWNTs in Organic Solvents

The sample of B1@*f*MWNTs was ultrasonicated with 20 mL of THF 1 h and the mixture was allowed to stand 24 h to settle any insoluble material. The precipitation was dried in an oven at 80 °C for 24 h and then weighed to determine the mass of solubilized MWNTs. Nine different concentrations of B1@*f*MWNTs in THF were obtained by diluting the supernatant from the above solution several times. The absorption spectrum of each of these solutions was then recorded, and the dependence of absorbance on concentration at 500 nm obeyed Beer’s law [24]. 

### 2.5. Fabrication of Epoxy Nanocomposites

The desired amount of bc@*f*MWNTs was dispersed in THF by ultra-sonication for 2 h at 40 °C and subsequently mixed with the epoxy resin by mechanical stirring and sonication for 30 min at 50 °C to obtain a black suspension. After completely removing the solvent by heating under a vacuum, the amount of epoxy resin and curing agent were added. The resin mixture was then placed in a planetary mixer and subjected to high-speed shear mixing and degassing steps. Then, the mixture was cast in preheated steel molds (60 °C) and cured at 80 °C for 120 min, 120 °C for 60 min, 140 °C for 60 min, and 160 °C for 120 min.

### 2.6. Characterizations

The number-average molecular weight (M_n_) and M_w_/M_n_ of the polymers were measured by gel permeation chromatography (GPC) equipped with Agilent 1100 GPC at room temperature, using THF as the eluent at a flow rate of 1 mL min^−1^. The UV–vis absorption spectra of bc@*f*MWNTs organic resolvent solutions, ranging from 350 to 750 nm, were measured with a UV–vis absorption spectrophotometer (Evolution 220, Thermo Scientific, Waltham, MA, USA). Thermogravimetric analysis (TGA) of the fillers was conducted by TGA4000 (Perking Elmer Instrument, Waltham, MA, USA) at a heating rate of 20 °C/min under a nitrogen atmosphere. Both transmission optical microscopy (TOM, ZEISS Axio Scope.A1) and transmission electron microscopy (TEM, Tecnai G220 electron microscope, Thermo Scientific, Waltham, MA, USA) were adopted to verify nanotubes’ dispersion in the matrix. An ultramicrotome (UCT-FCS Leica Microsystems, Austria) equipped with a diamond knife was used to cut the samples for TEM observation. Morphologies of the fracture surfaces of samples (after the tensile test) were sputtered with platinum and observed through scanning electronic microscopy (SEM) (Sirion 200, FEI Company, Eindhoven, The Netherlands).

### 2.7. Mechanical Test

The tensile test of Epoxy/MWNT composites was conducted on a universal testing machine (CMT4104, SANS, Shanghai, China) at a tensile rate of 5 mm min^−1^. The single edge notched bend (SENB) test was performed to measure the fracture resistance of epoxy/bc@*f*MWNT composites in mode I tension. The specimens were prepared according to the specifications, ASTM D5045-99, whose dimensions are 10 mm wide × 4 mm thick × 70 mm length. The SENB specimens were loaded in three-point bending at a crosshead speed of 10 mm/min and a span of 40 mm. For all mechanical tests, at least five samples of each blend were tested, from which the mean values and standard deviations were calculated. DMA properties were studied with a dynamic temperature ramp from 40 to 200 °C (ramping rate = 2 °C min^−1^) using a Q 800 analyzer (TA Instruments, New Castle, DE, USA), at 1 Hz with a constant strain of 20 mm by single cantilever mode. 

## 3. Results

### 3.1. Synthesis of Block Copolymers

All polymerizations proceeded with reasonable control, as evidenced by the low product polydispersity index (PDI) (Table 4). 

**Figure 1:**
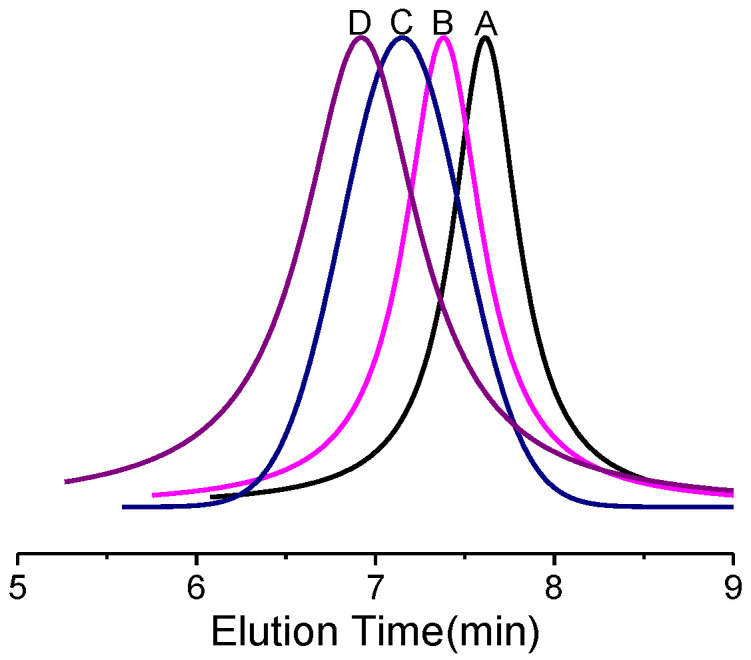
GPC curves of block copolymers (A) B1 (M_n_ = 10,700 g mol^−1^, M_w_/M_n_ = 1.12); (B) B2 (M_n_ = 15,150 g mol^−1^, M_w_/M_n_ = 1.13); (C) B3 (M_n_ = 19,500 g mol^−1^, M_w_/M_n_ = 1.12); (D) B4 (M_n_ = 27,300 g mol^−1^, M_w_/M_n_ = 1.15).

The GPC curve in Figure 1 shows the molecular weight and molecular weight distribution of the block copolymer prepared from the macro-CTA (PHMA), and no peak attributed to the starting macro-CTA (PHMA) was observed in the monomodal distribution curve, which meant the macro-CTA had been completely converted to the corresponding block copolymers. Furthermore, the block copolymers (Table 4) have reasonably low molecular weight distributions within 1.12–1.15. 

As expected, the monomer conversion was decreased from 88% to 40% with the DP_n_ of the macro-CTAs (PHMA) rising from 24 to 120 (Table 4). There are two reasons for the decreasing conversion. The first is that the macro-CTAs would sterically reduce the reactive probability between the living polymer chain end and the chain transfer unit. The second is the increasing viscosity of the mixture caused by the rise of molecular weight of the macro-CTAs, limiting the movement of the monomers. The polymerization of GMA was well controlled by changing the feed ratio of the monomer to the macro-CTA. Finally, the PGMA chains with a molecular weight of around 6000 g mol^−1^ were linked to the azido-terminated PHMA polymers. 

### 3.2. Preparation of bc@fMWNTs

**Figure 2:**
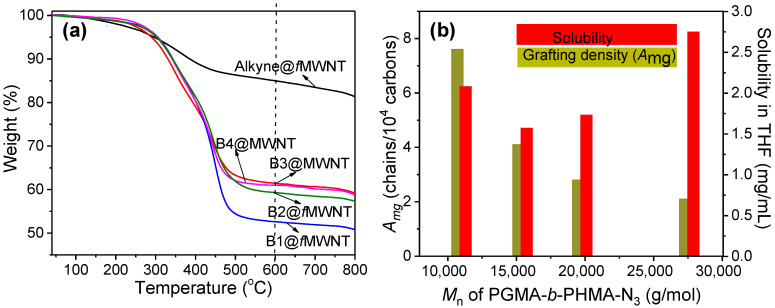
TGA curves (**a**) and solubility and grafting density (**b**) of bc@*f*MWNTs brushes with different PGMA-*b*-PHMA-N_3_ average molecular weights.

TGA measurements allow us to obtain the mass loss of the grafting of block copolymers on MWNTs under an N_2_ atmosphere. The curves for Alkyne@*f*MWNTs and bc@*f*MWNTs are displayed in Figure 2a. The grafted block polymers on the MWNT’s outer surface contribute to the increased weight loss between 200 and 600 °C. The grafting density of the polymers bound to the nanotubes is responsible for the disparity in weight loss (38.6~47.3%). The grafting density of copolymers was determined by Equation 1 based on the weight loss, and the molar mass of polymers leads to a value of 8.1~2.1 chains per 10,000 carbon atoms (see Figure 2). Obviously, as the molecular weight of the PHMA increases, the reactivity of the chain-end azide functionality decreases by the increasing chain entanglement [19]. As a result, the graft density dropped from 8.1 to 2.1 when the polymer molecular weight was increased from 10,700 to 27,300 g mol^−1^.

(1) Amg¯=10,000 MCWPMPWC (chains per 104 carbons)

### 3.3. Solubility of bc@fMWNTs

Solution-phase processing and manipulation are required to achieve homogeneous dispersions of block copolymer-modified carbon nanotubes within-host materials. So, it is reasonable to study the solubility of the bc@*f*MWNTs in various organic solvents. UV–vis spectroscopy [25,26] was used to investigate the absorption properties and the degree of solubility of bc@*f*MWNTs in this study. 

**Figure 3:**
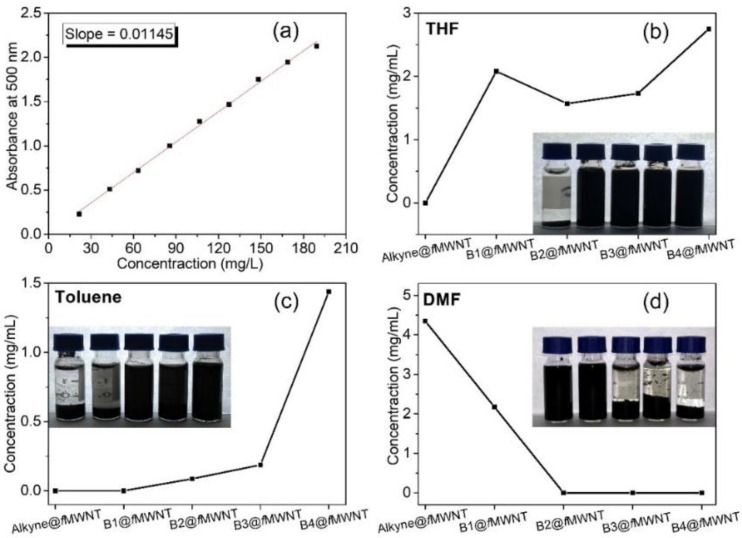
Optical density at 500 nm of bc@*f*MWNTs in THF at different concentrations. The straight line is a linear fit to the data having a slope of 0.01145. (Note: The concentration for UV–Vis measurement has been diluted several times) (**a**), and photograph, the solubility of different block copolymer modified MWNTs in THF (**b**), toluene (**c**), and DMF (**d**) after 24 h.

Nine concentrations of B1@*f*MWNTs in THF were obtained by diluting the supernatant from the above solution several times. The absorption spectrum of each of these solutions was then recorded, and the dependence of absorbance on concentration at 500 nm obeyed Beer’s law [24]. The absorption value of each solution at 500 nm was plotted against MWNTs concentration (Figure 3a). The slope of the linear-least-squares fit is then analogous to the familiar extinction coefficient, i.e., 0.01145, with an R-squared value of 0.99747: this value was used to determine the maximum concentration of the bc@*f*MWNTs in various organic solvents. To calculate the exact amounts of nanotubes present within the dissolved material used in the absorption experiment, TGA results (as shown in Figure 2a) were employed to determine the weight percent of nanotubes within bc@*f*MWNTs. The dispersion procedure was identical for all bc@*f*MWNTs.

Figure 3b–d show the solubility state of Alkyne@*f*MWNTs and the bc@*f*MWNTs in three types of organic solvents. THF is a suitable solvent for both PHMA and PGMA. Toluene is suitable for PHMA, but not for PGMA. DMF is a suitable solvent for PGMA, but not for PHMA. Interestingly, the solubility of bc@*f*MWNTs decreased slightly with the extending of the PHMA polymer chain and then increased with further extending the polymer chain, which was inconsistent with the report that the solubility of nanocarbon would decrease with the extending of the grafted polymer chain length [27]. The slight decrease effect of the extending polymer chain on solubility is due to the decreasing grafting density, without a doubt. Furthermore, the increased solubility of the carbon nanotube, with a further extending chain length of the polymer, could be attributed to the good solubilizing strength of the polymer with increasing molecular weight [26]. For toluene, a pronounced increase in solubility was observed for increasing the molecular weight of PHMA, which was attributed to the excellent solubility of toluene for PHMA. On the contrary, a pronounced decrease in solubility in DMF was observed for bc@*f*MWNTs, as the extending the chain length of PHMA. bc@*f*MWNTs tended to precipitate from solution over 24 h. Such decreased dispersion of bc@*f*MWNTs is also attributed to the poor solubility of DMF of PHMA. In conclusion, we believe that the dispersity of bc@*f*MWNTs could be modulated by altering the structural component of the grafted polymers.

### 3.4. Dispersity of bc@fMWNTs in Epoxy

**Figure 4:**
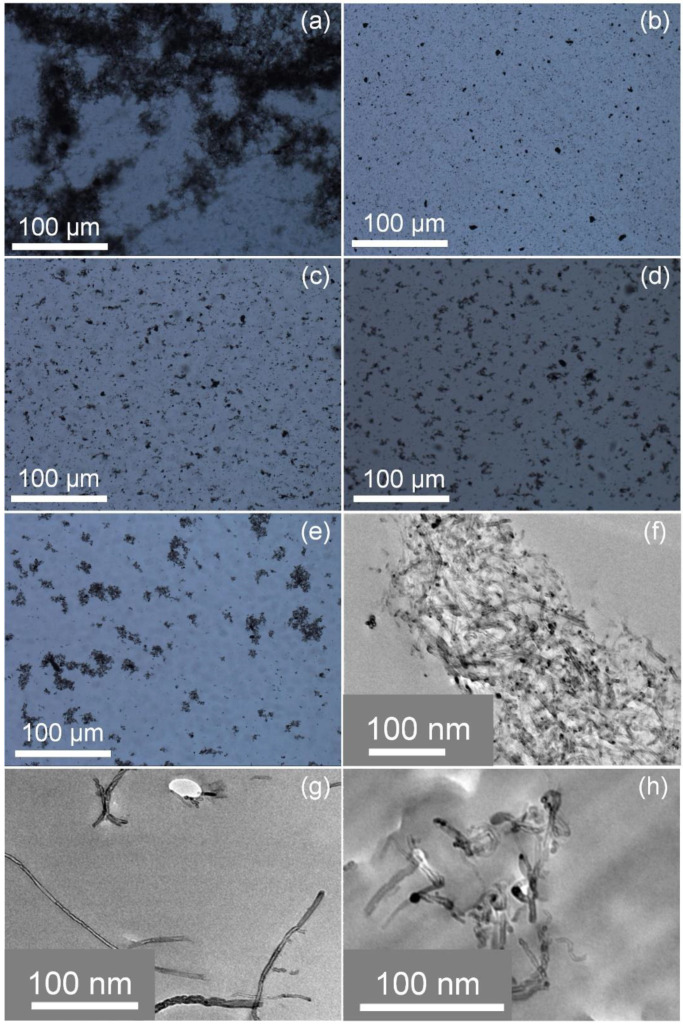
TOM images of the epoxy nanocomposites with 0.05 wt% (**a**) p-MWNTs, (**b**) B1@*f*MWNTs, (**c**) B2@*f*MWNTs, (**d**) B3@*f*MWNTs, (**e**) B4@*f*MWNTs, and TEM images of the epoxy nanocomposites with 0.05 wt%: (**f**) p-MWNTs, (**g**) B1@*f*MWNTs, (**h**) B4@*f*MWNTs.

It was found that nanofillers tend to re-agglomerate during the curing reaction because of the weak interaction [28,29] as the dispersibility of MWNTs in the epoxy is different from that in organic solvents. So, it is necessary to check the chain length effect of the grafted polymers on the dispersity of functionalized MWNTs in epoxy resin after the curing reaction. THF, the good solubility for both blocks of copolymers of bc@*f*MWNTs, was chosen as a solvent to disperse bc@*f*MWNTs in the epoxy. After block copolymers modification, the carbon nanotubes present a pronounced better dispersion than pristine MWNTs in the matrix (Figure 4). 

The dramatically improved dispersion level was attributed to the out-layer PGMA segment in bc@*f*MWNTs, providing good compatibility with the epoxy matrix. With the extending of the chain length of the inter-layer PHMA, the bc@*f*MWNTs dispersion level obviously deteriorated. The reason for this is the incompatibility of the inter-layer PHMA with epoxy. It is pleasing that the dispersion state of B4@*f*MWNTs is still better than that of p-MWNTs. We believe that the PGMA segment is vital to ensure the better dispersibility of nanotubes in the epoxy in this system. The TEM graphs of epoxy/bc@*f*MWNTs composites could demonstrate this phenomenon. Compared with epoxy/p-MWNT composites, MWNTs in epoxy/B1@*f*MWNT composites (Figure 4g) and epoxy/B4@*f*MWNT composites (Figure 4h) were dispersed more uniformly. More tightly wrapped MWNTs were observed in epoxy/B4@*f*MWNT composites than epoxy/B1@*f*MWNT composites.

### 3.5. Dynamic Mechanical Properties of Epoxy/bc@fMWNTs

**Figure 5:**
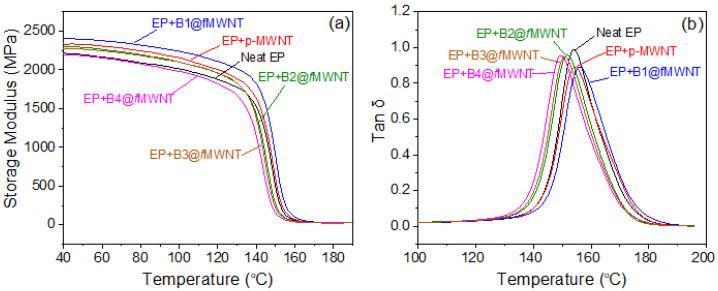
Storage modulus (**a**) and tan δ (**b**) of epoxy composites with 0.05 wt% of p-MWNTs and bc@MWNTs.

As mentioned before, the inter-layer PHMA segment in bc@*f*MWNTs can tailor the dispersion level and the interface between MWNTs and epoxy. The effect of the chain length of PHMA on the storage modulus and glass transition temperature (*T*_g_) of the epoxy nanocomposites were examined by dynamic mechanical analysis. Figure 5 depicts the storage modulus and tan delta curves as a function of temperature for the cured neat epoxy and various composites. As shown in Figure 5a, due to the softening of the polymer chains with temperature, the storage moduli of the neat epoxy and its composites are found to decrease with temperature [30]. It is well known that the introduction of rubber into the epoxy/nanofiller composite would reduce the storage modulus of the composite due to the increased flexibility of polymer chains [31]. However, the composite with B1@*f*MWNTs showed an increased storage modulus (2405.6 MPa) compared to the neat epoxy (2220.8 MPa) in the glassy state at 40 °C, whereas adding 0.05 wt% pristine MWNTs to epoxy induced a slight increase in storage modulus (2329.3 MPa). This slight increase can be explained by the uniform dispersion and the strong interfacial bonding between B1@*f*MWNTs and the epoxy provided by the epoxy groups on the side chain of PGMA. It is reasonable to suppose that the load gets transferred from the epoxy matrix to the filler, resulting in the enhanced modulus [32]. As shown in Figure 3a, the storage modulus of the epoxy composites decreased lightly with the increasing molecular weight of inter-layer PHMA because of the rubbery nature of PHMA, which would inevitably reduce the storage modulus of the composite [33]. 

The temperature at which the loss factor curve showed a maximum peak is often recorded as the *T*_g_. From Figure 5b, it is seen that a small amount of p-MWNTs and bc@*f*MWNTs have a different influence on the *T*_g_ values of the epoxy resin. For example, the *T*_g_ values for the composite with the poorly dispersed pristine MWNTs are slightly shifted to a higher temperature than the neat epoxy (from 153.9 °C to 154.3 °C). B1@*f*MWNTs further enhanced the *T*_g_ value to 156.2 °C. Then, the *T*_g_ values for the composite decrease slightly with a further increase in molecular weight of the PHMA segment (151.6 °C for epoxy/B2@*f*MWNT composites, 151.1 °C for epoxy/B3@*f*MWNT composites, and 149.7 °C for epoxy/B4@*f*MWNT composites). This phenomenon can be attributed to the following factors: (i) dispersion level of MWNTs in epoxy resin; (ii) tangled structure and large surface area of MWNTs; (iii) covalent bonding between the MWNTs and the epoxy resins; and (iv) molecular structure of grafted block copolymers and cross-linking degree of curved epoxy composites. These competing factors determine the change of *T*_g_ values. The tangled structures and large surface area likely inhibit epoxy chains’ mobility, effectively increasing the *T*_g_ value of materials. Additionally, the outer-layer reactive PGMA grafted on the MWNTs provides covalently interfacial bonding between MWNTs and the epoxy polymer chain, leading to the polymer’s confinement in the nanotubes’ vicinity. On the contrary, the poor dispersion of nanotubes would weaken the efficiency in restricting the mobility of the surrounding polymer [34]. The flexible PHMA grafted on the MWNTs could produce soft chains at the matrix/nanotube interface and thus reduce the confinement effect on the epoxy molecular chains. The outer layer PGMA grafted on MWNTs would partially react with the curing agent. The microstructure of epoxy cross-linked networks around the epoxy-rich surfaces is changed, reducing the degree of cross-linking and even leading to a drop in *T*_g_ value. Therefore, the increase in *T*_g_ value can be attributed to the better dispersion and improved interfacial adhesion between B1@*f*MWNTs and epoxy resin [15,35]. However, as the inter-layer PHMA chain extends, the wrapped morphology of nanotubes (see Figure 2) and softer chains at the nanotube/matrix interface reduce the confinement effect on the epoxy molecular chains [34]. 

### 3.6. Mechanical Properties

**Figure 6:**
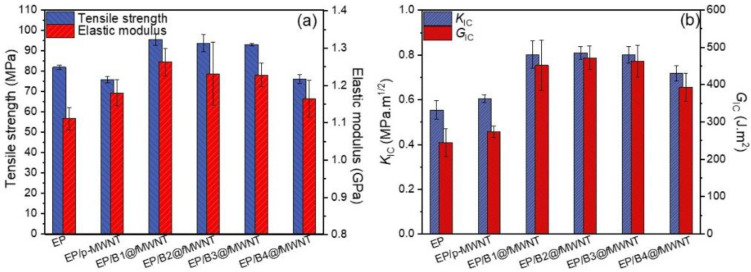
Tensile properties (**a**) and fracture toughness (**b**) of epoxy-based nanocomposites.

In this work, PGMA-*b*-PHMA with different PHMA molecular lengths and the same PGMA molecular length were grafted on the MWNTs surface, enabling the carbon nanotubes to link with epoxy networks covalently. We attempted to study how the structural change of PHMA in bc@*f*MWNTs influences the mechanical properties of resulting composites. The tensile properties and fracture toughness for the neat epoxy and its composites with different fillers are shown in Figure 6. At the same loading of 0.05 wt%, all epoxy/bc@*f*MWNT composites exhibit higher tensile properties and fracture toughness than epoxy/p-MWNT composites. 

**Figure 7:**
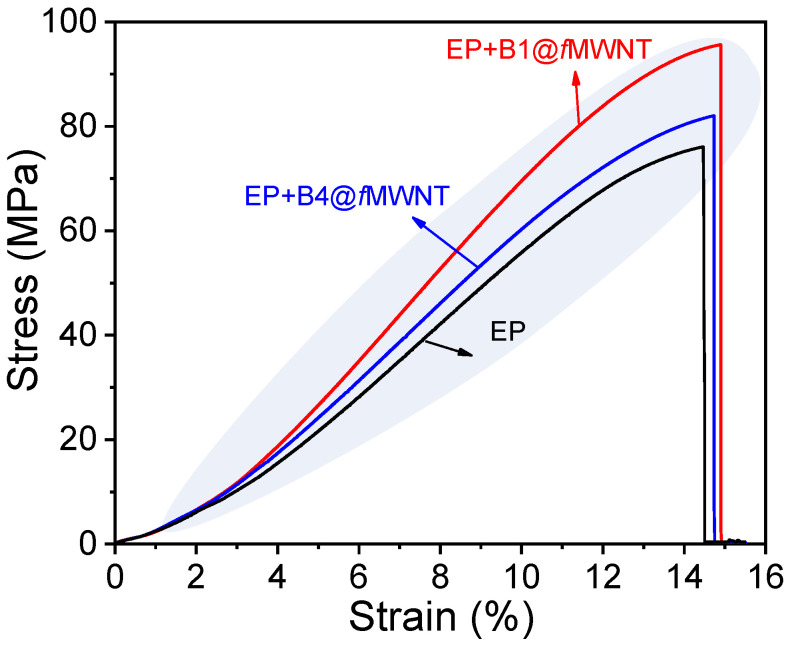
Stress–strain curves of epoxy, epoxy composites with 0.05 wt% B1@*f*MWNTs and B4@*f*MWNTs.

Representative stress–strain curves for the neat epoxy and its nanocomposites are plotted in Figure 7, along with the results of uniaxial tensile testing. The introduction of pristine MWNTs negatively affects tensile strength and modulus due to the big aggregate (see Figure 4) and weak epoxy–nanotube interface. On the contrary, it can be clearly seen that the introduction of B1@*f*MWNTs, B2@*f*MWNTs, and B3@*f*MWNTs produce dramatically improved strength and toughness, along with a similar increase in stiffness. For the neat epoxy, the ultimate tensile strength is 82.0 MPa. At 0.050 wt% B1@*f*MWNTs, B2@*f*MWNTs, and B3@*f*MWNTs, the maximum tensile strengths of nanocomposites are 95.6 Mpa, 93.8 Mpa, and 93.1 Mpa, corresponding to the increases of 16.6%, 14.4%, and 13.5%, respectively. The tensile strength of bc@*f*MWNTs-filled epoxy composites slightly reduces as the molecular weight of PHMA reaches 8250 g mol^−1^ (B2@*f*MWNTs). The minor average tensile strength of cured epoxy composites is 76.1 Mpa, with B4@*f*MWNTs higher than the epoxy/p-MWNT composite (75.9 Mpa). The modulus of epoxy nanocomposites with 0.05 wt% bc@*f*MWNT show a lighter decrease as a function of molecular weights of PHMA increase from 4480 to 20,800 g mol^−1^ than the decrease in strength.

**Figure 8:**
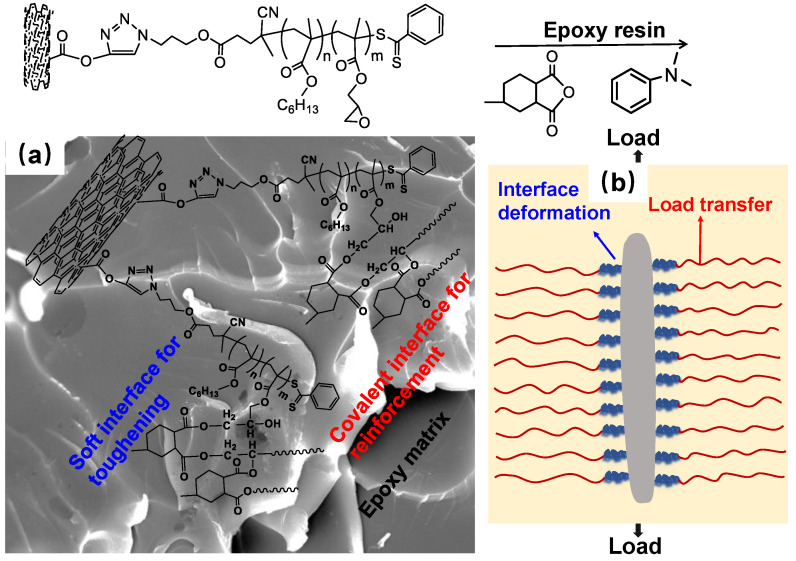
Proposed epoxy/bc@*f*MWNTs nanocomposite formation mechanism (**a**), interphase structures around MWNTs and their effects upon loading (**b**).

As illustrated in Figure 8, the PHMA and PGMA molecules interact differently with the epoxy chain. On the one hand, the increased compatibility and chemical interaction between bc@*f*MWNTs and epoxy, provided by the segment of PGMA, constrains the mobility of segments, and thus contributes to a load well transfer at the interface. On the other hand, due to the longer linear molecular length, the extending PHMA chains on the MWNTs can suffer from the worse dispersion state, confirmed by OM, and significantly higher deformation through the soft and flexible interphase. The worse dispersion and higher deformation are unfavorable for enhancing the load transfer in the host polymer. Hence, the tensile strength and modulus decrease as a function of the molecular weights of PHMA which increases from 4480 to 20,800 g mol^−1^. It is well-known that the modulus is dependent on the modulus and volume fraction of the composite constituents. Thus, there is a smaller decrease in a function of molecular weights of PHMA, in the case of modulus compared to strength. 

The fracture toughness values (*K*_IC_ and *G*_IC_) of the neat epoxy and its composites with 0.05 wt% different fillers are listed in Table 1. For the given specimen geometry, Poisson’s ratio ( ν) of the neat epoxy (i.e., 0.35 [36]) is used to determine the value of *K*_IC_. The *K_IC_* of nanocomposites with p-MWNTs increased by ~10%, compared to the base value of neat epoxy. In comparison, the presence of bc@*f*MWNTs in nanocomposites results in a remarkable increase in fracture toughness. Especially, Epoxy/B1@*f*MWNT composite and Epoxy/B2@*f*MWNT composite show a sharp increase in *K*_IC_, followed by a trend in a slight decrease with longer PHMA chains. The composites with 0.05 wt% B2@*f*MWNTs exhibit maximum toughening effects, for which *K_IC_* and *G_IC_* increase by 46% and 92.6%, respectively. The abovementioned results show significant improvements in toughness, indicating the tunable and effective interphases obtained between the nanotubes and the matrix. At this moment, how the different PGMA-*b*-PHMA chains differ in influencing fracture toughness remains an open question. It is well-known that epoxide groups on the MWNTs surface provide good dispersion of bc@*f*MWNTs in the epoxy and chemical bonding between MWNTs and the matrix. Moreover, the PHMA chains with different chain lengths should create different soft interphase structures between the MWNTs and bulk epoxy, influencing the overall performance of nanocomposites [37]. Hence, the longer PHMA chains on the surface of MWNTs would afford higher deformation for the matrix and an enhanced mobility for MWNTs because of the soft and flexible nature of PHMA, enhancing the energy dissipation during deformation. However, as the polymer’s molecular weight of PHMA increases, the dispersion level of bc@*f*MWNTs in the epoxy declines (i.e., the size of the aggregates gradually increases from Epoxy/B1@*f*MWNTs to Epoxy/B4@*f*MWNTs) (Figure 2). This phenomenon is harmful to the toughening effect of composites [38,39]. Summarizing the abovementioned results, incorporating the PHMA chains onto MWNT’s surface to form MWNT-based molecular brushes with different chain lengths can have two competitive and opposing effects on the fracture toughness of the composite. A balance between dispersion state and soft interface must be reached to maximize the toughness of composites.

To put the mechanical properties‘ results in the perspective of published studies of tensile strength and fracture toughness in epoxy/CNT nanocomposites, we find that the introduction of organic molecules in the functionalized CNTs improved either tensile strength or fracture toughness to a certain extent at 0.2–1 wt% loadings [40,41]. In this work, bc@*f*MWNT only required loading of 0.05 wt% to obtain a similar improvement in strength, K*_IC_*, or G*_IC_*, indicating our interfacial design works. 

**Figure 9:**
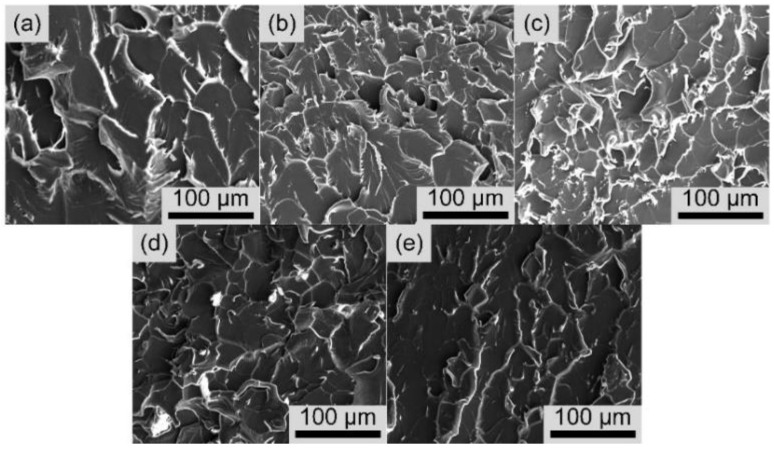
SEM fractographs of the epoxy nanocomposites with 0.05 wt%: (**a**) p-MWNTs, (**b**) B1@*f*MWNTs, (**c**) B2@*f*MWNTs, (**d**) B3@*f*MWNTs, (**e**) B4@*f*MWNTs.

As shown in Figure 9, the fracture surface composites, filled with p-MWNTs and bc@*f*MWNTs taken from tensile tests, were investigated by SEM to further research the fracture characteristics of the epoxy composites. The fracture surface of all samples shows dimple-like structures on the fracture surface. At the same time, the composites filled with bc@*f*MWNTs (Figure 9e) are covered with more dimple-like structures than p-MWNTs (Figure 5a), indicating that the interfacial adhesion between bc@*f*MWNTs and epoxy resin has been improved. This enhanced interfacial interaction is beneficial for the load effectively transferring from the epoxy to MWNTs [42], resulting in a reinforcing effect of its composites. The composites filled with B1@*f*MWNTs (Figure 5b) and B2@*f*MWNTs (Figure 9c) show the most dimple-like structures on the fracture surface, while those loaded with B4@*f*MWNTs (Figure 9e) show the least of that kind of structure. The creation of new fracture surfaces accompanies the formation of dimple-like structures, and thus considerable fracture energy is likely to be dissipated [43]. These results agree with the abovementioned mechanical performance (Figure 6).

## 4. Conclusions

In this paper, we demonstrated the interfacial effects of grafted PGMA-*b*-PHMA chain lengths on the mechanical properties of epoxy/bc@*f*MWNT composites. A series of azide-terminated PGMA-*b*-PHMA copolymers with different PHMA chain lengths and similar PGMA chain lengths were grafted on the surface of MWNTs. TGA thermograms of bc@*f*MWNTs indicate that the grafting density of PGMA-*b*-PHMA chains grafted on MWNTs decrease with an increae in the molecular weight of block copolymers. UV–vis spectroscopy demonstrates that the different chain lengths of PHMA on bc@*f*MWNTs have a tremendous impact on the solubility of MWNTs in various organic solvents. Images from OM and TEM of the nanocomposites demonstrate that the nanocomposites containing bc@*f*MWNTs with longer grafted PHMA chains harmed the dispersion of MWNTs in an epoxy matrix because of the incompatible nature between epoxy and PHMA. The SEM micrographs of the nanocomposites prove the improved interfacial quality between functionalized MWNTs and epoxy hosts. The epoxy/bc@*f*MWNT composites showed better tensile and fracture toughness properties than neat epoxy and epoxy/p-MWNT composites. Interestingly, the tensile strength and modulus decrease as a function of molecular weights of PHMA increases from 4480 to 20,800 g mol^−1^. Epoxy/bc@*f*MWNT composites with relatively short PHMA chains (i.e., less than 10,000 g mol^−1^) show a sharp increase in fracture toughness, followed by a slight decrease with longer PHMA chains. The related mechanisms of the epoxy/bc@*f*MWNT composites are because longer PHMA chains cause the two competitive and opposing effects on the dispersion state and soft interface, which must be optimized to maximize the mechanical properties of composites.

## Figures and Tables

**Table 1:** Properties of epoxy resin E51.

The Qualitative Characteristics of E51	Value
Viscosity (25 °C, Pa·s)	12–15
Epoxy Value (eq/100 g)	0.51–0.53
Epoxide equivalent weight (g/1 eq)	191–193
Density at 25 °C (g/cm^3^)	1.16

**Table 2:** Properties of hardener.

Name	Viscosity (25 °C, mPa·s)	Density at 25 °C (g/cm^3^)
MHHPA	40~50	1.16
BDA	90	0.9
Mixture (40:1)	40–50	1.15

**Table 3:** Properties of MWNTs.

The Qualitative Characteristics of MWNTs	Value
Internal diameter (nm)	8−15
External diameter (nm)	15–20
Length (μm)	0.5–2
Total amount of admixtures (%)	≤1

**Table 4:** RAFT polymerization of HMA and GMA in dioxane.

Code	CTA	[M]:[CTA]:[I]	Con (%)	M_n_,_PHMA_^a.^	M_n_, _PGMA_^c.^	M_n_,_PHMA-*b*-GMA_^c.^	PDI ^a.^
H1	CTA-N_3_	25:1:0.2	90	4480	−	−	1.08
H2	CTA-N_3_	50:1:0.2	94	8250	−	−	1.09
H3	CTA-N_3_	90:1:0.2	85	13,800	−	−	1.07
H4	CTA-N_3_	180:1:0.2	70	20,800	−	−	1.10
B1	PHMA_24_-N_3_^b.^	35:1:0.2	88	4480	6290	10,700	1.12
B2	PHMA_46_-N_3_ ^b.^	50:1:0.2	85	8250	6900	15,150	1.13
B3	PHMA_79_-N_3_^b.^	85:1:0.2	49	13,800	5700	19,500	1.12
B4	PHMA_120_-N_3_ ^b.^	95:1:0.2	41	20,800	6500	27,300	1.15

^a^ Determined by gel permeation chromatography (GPC) in THF relative to monodispersed polystyrene standards. ^b^ calculated by (M_n,PHMA_-M_CTA_)/M_HMA_, where M_CTA_ = 362, M_HMA_ = 142, M_n,PHMA_ = 4480, 8250, 13,800, and 20,800 g mol^−1^, respectively. ^c^ M_n,GMA_ = M_n,PHMA-*b*-GMA_-M_n,maro-CTA._

## Data Availability

The data presented in this study are available on request from the corresponding author.
